# Circ_0001667 promotes breast cancer cell proliferation and survival via Hippo signal pathway by regulating TAZ

**DOI:** 10.1186/s13578-019-0359-y

**Published:** 2019-12-30

**Authors:** Zhongli Geng, Wei Wang, Hui Chen, Jianya Mao, Zhenguo Li, Jing Zhou

**Affiliations:** 1 Department of Surgery, Affiliated Hospital of Traditional Chinese, Medicine Xinjiang Medical University, 116 Huanghe Road, Urumchi, 830000 Xinjiang China; 2 Department of Head and Neck Surgery, Qitai County People’s Hospital, Changji, Xinjiang China

**Keywords:** Breast cancer, circ_0001667, miR-125a-5p, TAZ, Hippo pathway

## Abstract

**Background:**

Breast cancer is a most common type of cancer in women. Circular RNAs (circRNAs) are involved in cancer development and progression, but their roles and regulatory mechanisms are unclear in breast cancer. Our previous study indicated that has_circ_0001667 (circ_0001667) was up-regulated in breast cancer from the array and might play an oncogenic role, however, the roles of circ_0001667 were not known. This study was aimed to investigate the role and the underlying molecular mechanism of circ_0001667 in breast cancer.

**Results:**

The real-time PCR result showed that circ_0001667 was overexpressed in breast cancer tissues or cell lines compared to the adjacent normal tissues or normal cells. There was a negative relationship between circ_0001667 levels and the life time of breast cancer patients. Meanwhile, the inhibition of circ_0001667 suppressed the proliferation and metastasis of human breast cancer cells. Further bioinformatical analysis indicated that circ_0001667 sponged miR-125a-5p to regulate TAZ expression by Targetscan and miRanda. Dual luciferase reporter assay and western blotting experiments revealed that circ_0001667 negatively regulated miR-125a-5p expression leading to promoting TAZ expression through Hippo signal pathway in breast cancer cells.

**Conclusions:**

This study uncovered that circ_0001667 was a potential breast cancer prognostic marker, as well as a potential therapeutic target to inhibit breast cancer metastasis by circ_0001667/miR-125a-5p/TAZ axis.

## Background

Breast cancer is by far the most common malignant neoplasm in women worldwide [1]. At the present, the main therapeutic methods for breast cancer include surgery, chemotherapy and radiotherapy. Although some research has uncovered a better understanding and diagnosis of breast cancer, the underlying molecular and cellar mechanisms of breast cancer are still far from clear [2]. It is very important to unravel the potential mechanism on breast cancer progression and metastasis in order to find new targets for clinical therapy [3].

Circular RNAs (circRNAs), a type of non-coding RNA, have no 5′-3′ polarity and polyA tail and play an important role in regulating gene expression through competitive binding miRNAs [4]. Many studies confirmed that circRNAs are involved in the progression and development of breast cancer by regulating gene expression. For example, Liang et al. [5] found that circ-ABCB10 promoted breast cancer proliferation and progression through sponging miR-1271. Tang et al. [6] revealed that hsa_circ_0001982 knockdown suppressed breast cancer cell proliferation and invasion and induced apoptosis by targeting miR-143. Liang et al. [7] clarified CircDENND4C was a HIF1α-associated circRNA promoting the proliferation of breast cancer cells under hypoxia. Therefore, it is necessary to explore the critical role of circRNAs in breast cancer.

The Hippo pathway, highly conserved from drosophila to mammals, regulates cell proliferation, growth and apoptosis [8, 9]. Moreover, the Hippo pathway also controls organ size, tissue homeostasis, and stem cell self-renewal [10, 11]. The deregulation of the Hippo signaling pathway is observed in many human cancers, which play a key role in tumor initiation and progression [10, 12–14]. The mammalian Hippo pathway consists of a core kinase cascade including Mst1/2, LATS1/2 and downstream protein YAP (Yes-associated protein) and TAZ (transcriptional co-activator with PDZ binding motif) [15]. Once the Hippo pathway is activated, Mst1/2 combines with adaptor protein WW45 to form a complex compound and then phosphorylates the LATS1/2 kinases and another adaptor protein MOB to compose LATS/MOB complex [10, 16, 17]. Subsequently, the LATS/MOB complex active and phosphorylate the downstream protein YAP and its paralog TAZ, leading to cytoplasmic retention and proteasome-mediated degradation [12, 18].

Our previous study indicated that hsa_circ_0001667 (circ_0001667) was up-regulated in breast cancer from the array and might play an oncogenic role, however, the regulatory roles of circ_0001667 were not known. The study will verify the expression of circ_0001667 and investigate the molecular role and mechanism of circ_0001667 in breast cancer. Our study demonstrated that the circ_0001667 levels was significantly higher in breast cancer tissues than adjacent normal tissues. Conversely, an inverse expression of miR-125a-5p sponged with circ_0001667 was observed. Moreover, we revealed that circ_0001667 inhibition decreased breast cancer cell proliferation and metastasis and activated the Hippo pathway by regulating TAZ.

## Results

### Circ_0001667 expression was up-regulated and related to the shorten life time in breast cancer

**Fig. 1:**
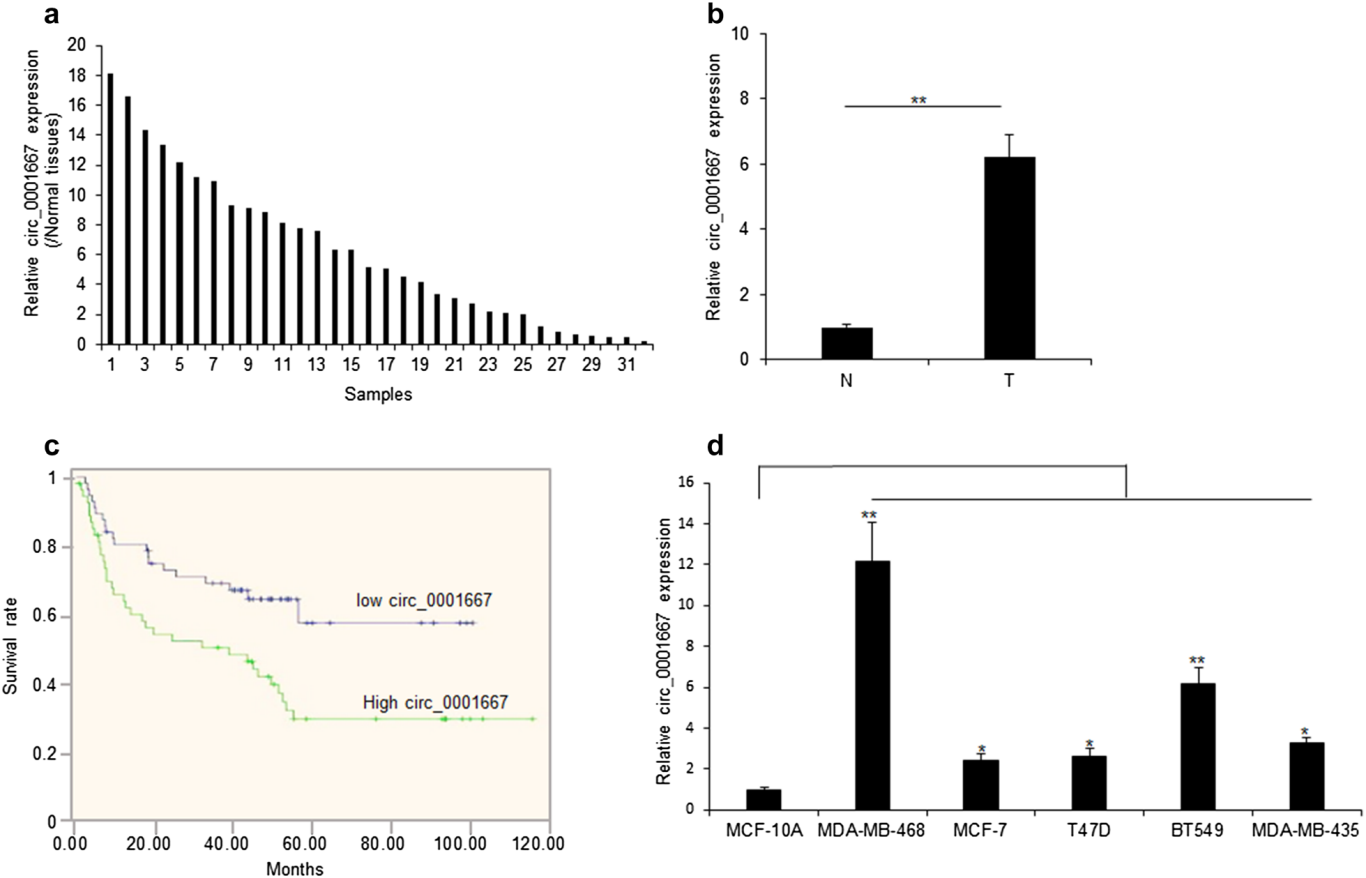
Up-regulation of circ_0001667 expression in breast cancer tissues was related to the shorten life time. **a** Relative circ_0001667 expression levels in breast cancer tissues comparing to its expression in normal tissues. *N* normal tissues, *T* breast cancer tissues. Circ_0001667 expression was measured by real time RT-PCR. **b** Data analysis from **a**. **c** Circ_0001667 levels in breast cancer was negatively related to breast cancer patients’ life time. **d** Circ_0001667 expression was higher in breast cancer cell lines than it in breast epithelial cells (MCF-10A). *p < 0.05; **p < 0.01

To investigate the role of circ_0001667 in breast cancer, the expression of circ_0001667 in 32 pairs of human breast cancer tissues from the patients were measured. The age range of the patients were 32–76 ages. 21 patients were ductal breast cancer and 11 patients were invasive ductal breast cancer without chemotherapy and radiotherapy. Our data showed that circ_0001667 expression was up-regulated significantly in breast cancer tissues when compared to non-tumor tissues (Fig. 1a, b). Moreover, we also explored the relationships between circ_0001667 expression and the life time of breast cancer patients. We found that breast cancer patients with high levels of circ_0001667 had poor survival ability compared to the patients with low circ_0001667 expression (Fig. 1c). In addition, the circ_0001667 expression was tested in breast cancer lines including MCF-10A, MDA-MB-468, T47D, BT459 and MDA435. MCF-10A was a normal breast cancer cell line as control. The result revealed that circ_0001667 expression was higher in breast cancer cell lines than in MCF10A cells (Fig. 1d).

### Circ_0001667 inhibition decreased breast cancer cell proliferation and metastasis

**Fig. 2:**
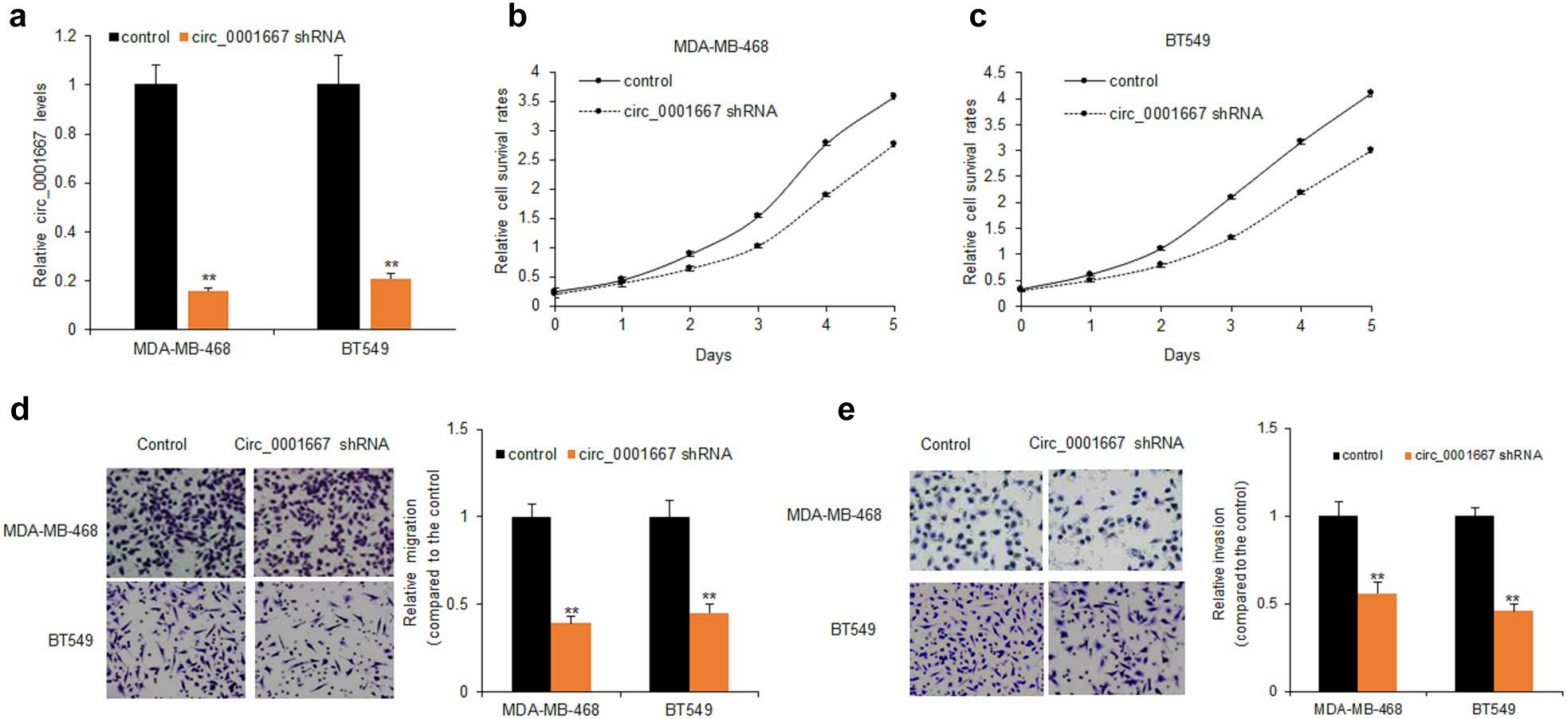
Circ_0001667 inhibition decreased breast cancer cell proliferation and metastasis. **a** Circ_0001667 expression in MDA-MB-468 and BT549 cells. MDA-MB-468 and BT549 cells were infected with lentivirus with circ_0001667 shRNA for 48 h and total RNA was extracted for real time RT-PCR. **b** The survival ability in MDA-MB-468 cells with circ_0001667 down-regulation. MDA-MB-468 cells were infected with lentivirus with shRNA and the cell proliferation was assayed by CCK8 from day 0 to day 5. **c** The survival ability in BT549 cells with circ_0001667 down-regulation. MDA-MB-468 cells were infected with lentivirus with shRNA and the cell proliferation was assayed by CCK8 from day 0 to day 5. **d** The migration ability in MDA-MB-468 cells with circ_0001667 down-regulation. MDA-MB-468 and BT549 cells were infected with lentivirus with shRNA and the cell migration was assayed by transwell system. **e** The invasion ability in MDA-MB-468 cells with circ_0001667 down-regulation. MDA-MB-468 and BT549 cells were infected with lentivirus with shRNA and the cell migration was assayed by transwell chamber with Matrigel treatment. **p < 0.01

To study the role of circ_0001667 in breast cancer, circ_0001667 shRNA was introduced into MDA-MB-468 and BT549 cells by transfection and we examined the circ_0001667 expression in breast cancer cells with circ_0001667 shRNA transfection using lentivirus. As shown in the Fig. 2a, the expression of circ_000167 was dramatically decreased in cells with circ_0001667 shRNA transfection (Fig. 2a). To further explore the functions of circ_0001667 in breast cancer cells, MDA-MB-468 and BT549 cells were transfected with circ_0001667 shRNA for cell survival ability assay. The data from CCK8 assay showed that the proliferation was significantly decreased in MDA-MB-468 and BT549 cells (Fig. 2b, c). Consistently, we also tested the migration and invasion ability of MDA-MB-468 and BT549 cells with circ_0001667 down-regulation. The representative pictures from the migration assay indicated that the migrated cell number was significantly decreased in MDA-MB-468 and BT549 cells with circ_0001667 down-regulation when comparing to the controls (Fig. 2d). The similar result was observed in invasion of breast cancer cells (Fig. 2e). These data uncovered that down-regulation of circ_0001667 inhibited proliferation and metastasis of breast cancer cells.

### Prediction for circ_0001667/miRNA interactions and miRNA-mediated signaling pathways

**Fig. 3:**
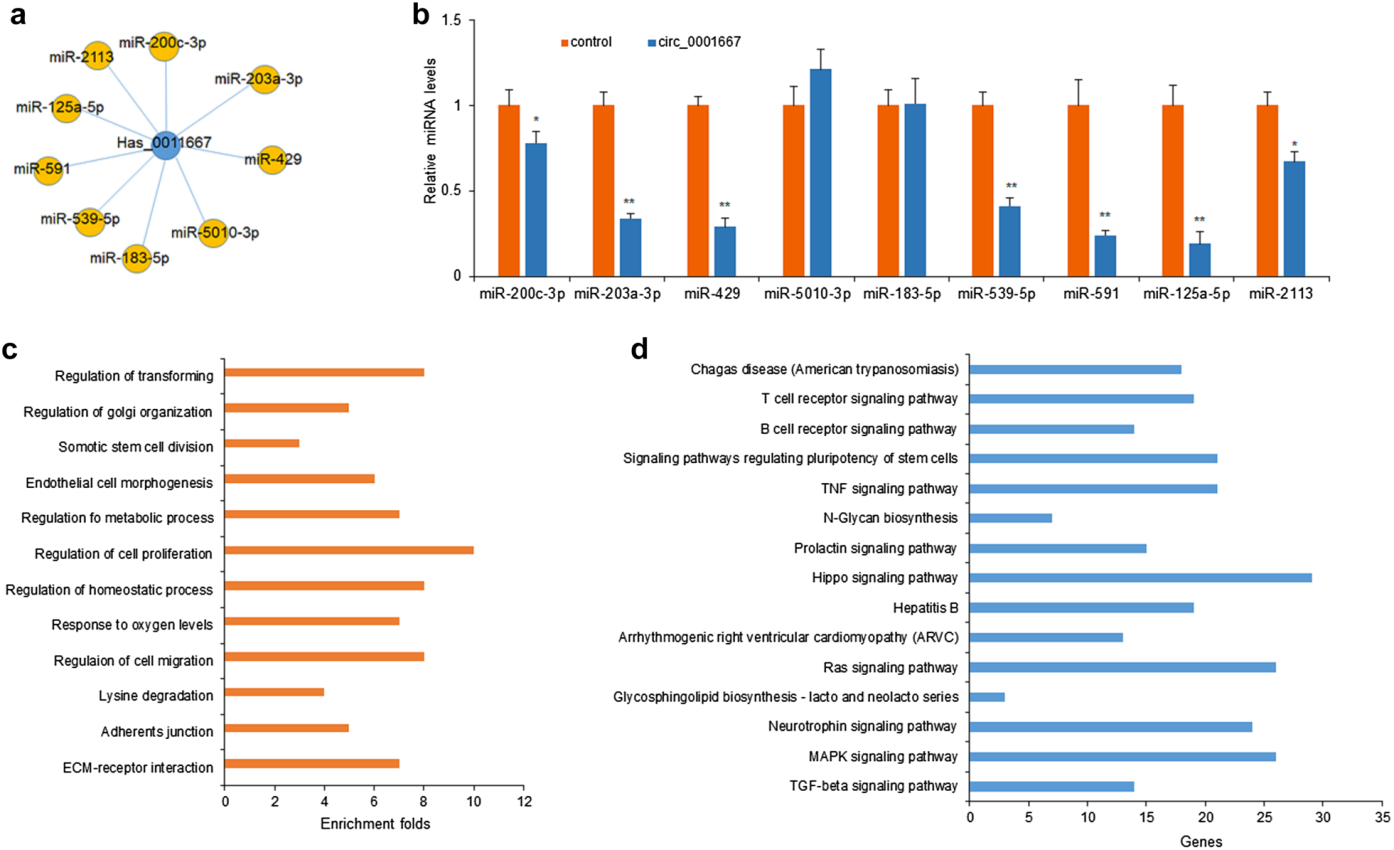
Prediction for circ_0001667/miRNA interactions and miRNA-mediated signaling pathways. **a** The interaction of circ_0001667-miR-125a-5p was predicted based on TargetScan and miRanda. **b** MiRNA expression in MDA-MB-468 cells. The miRNAs including miR-200c-3p, miR-203a-3p, miR-429, miR-5010-3p, miR-183-5p, miR-539-5p, miR-591, miR-125a-5p and miR-2113 were tested in MDA-MB-468 cells with circ_0001667 up-regulation by transfection. **c** Gene Ontology (GO) enrichment analysis of the target genes corresponding to circ_0001667. **d** Kyoto Encyclopedia of Genes and Genomes (KEGG) pathway analysis of the target genes showing the upregulated circ_0001667-related pathways. **p < 0.01

CircRNAs are important biological regulators by sponging miRNAs [19]. To explore the miRNA sponges by circ_0001667, the potential sponges of circ_0001667 were predicted Circnet (Fig. 3a). The miRNAs including miR-200c-3p, miR-203a-3p, miR-429, miR-5010-3p, miR-183-5p, miR-539-5p, miR-591, miR-125a-5p and miR-2113 were tested in MDA-MB-468 cells and miR-125a-5p was down-regulated in cells with circ_0001667 overexpression (Fig. 3b). There was a high significance and were more genes in regulation of cell proliferation and migration in GO analysis, which were associated with circ_0001667 (Fig. 3c). The KEGG analysis showed that circ_0001667 might regulate breast cancer progression via Hippo signaling pathway (Fig. 3d). The data showed that miR-125a-5p mediated in the Hippo pathway.

### Circ_0001667 sponged miR-125a-5p in breast cancer cells

**Fig. 4:**
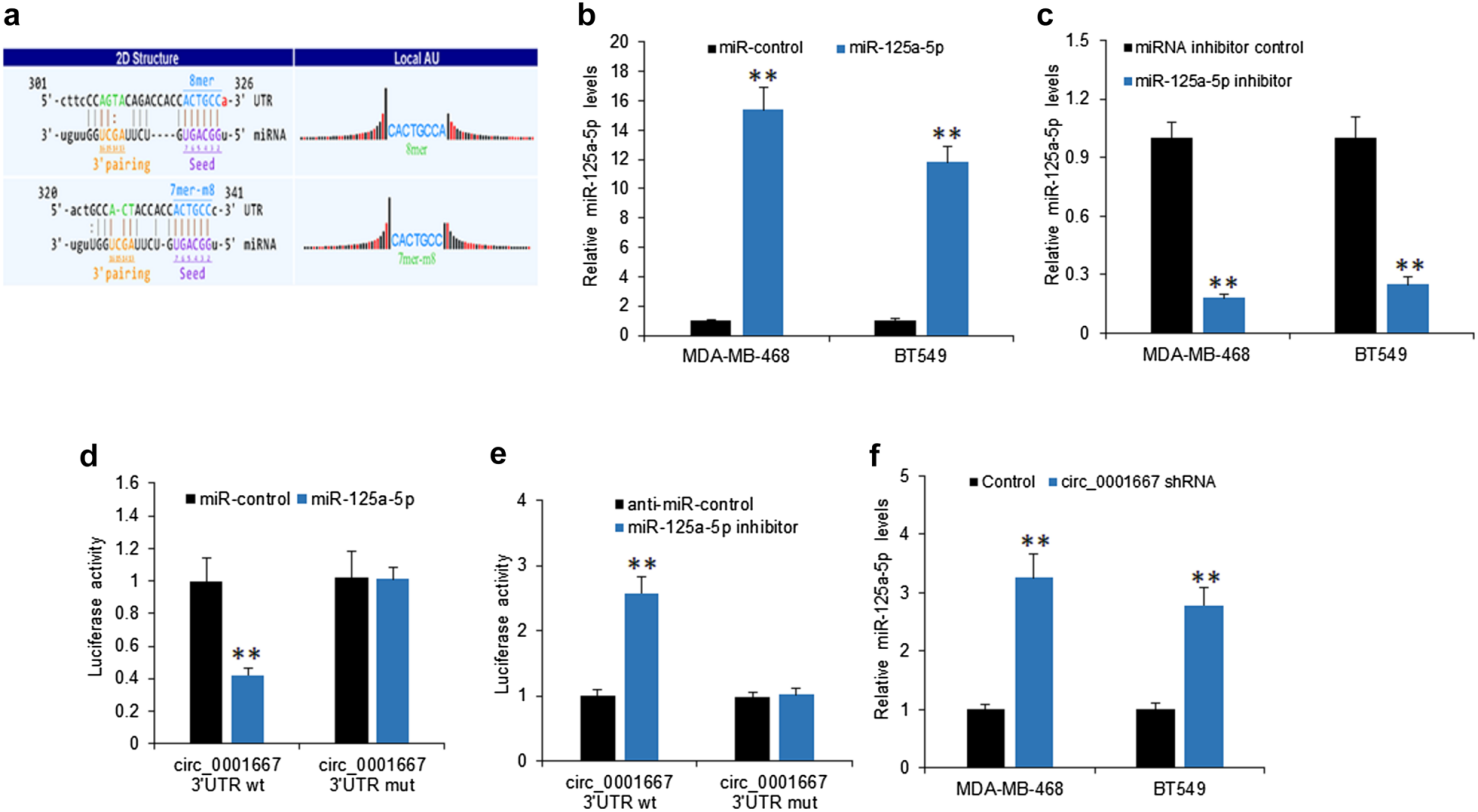
Circ_0001667 sponged miR-125a-5p in breast cancer cells. **a** The predicted binding sites of miR-125a-5p within circ_0001667 were shown. **b**, **c** MiR-125a-5p expression in MDA-MB-468 and BT549 cells. Cells were transfected with miR-125a-5p mimic or miR-125a-5p inhibitor and qRT-PCR analysis was used to detect the transfection efficiency. **d**, **e** Luciferase activity of the cells. MDA-MB-468 cells were cotransfected with miR-125a-5p mimics or miR-125a-5p inhibitor and luciferase reporter containing circ_0001667 3′-UTR (wt) or mutant construct (mut). The luciferase activity was assayed by luciferase assay. **f** MiR-125a-5p expression in MDA-MB-468 and BT549 cells with circ_0001667 down-regulation. Cells were transfected with circ_0001667 shRNA or the control, and the expression of miR-125a-5p determined by qRT-PCR. **p < 0.01

To determine the role of miR-125a-5p sponged by circ_0001667 in breast cancer cells, firstly, miR-125a-5p shared the complementary binding sites with circ-0001667 by bioinformatic prediction (Fig. 4a). Furthermore, we transfected miR-125a-5p mimic to MDA-MB-468 and BT459 cells, and qRT-PCR result showed that miR-125a-5p was over-expressed (Fig. 4b). Similarly, the expression of miR-125a-5p was significantly decreased when the miR-125a-5p inhibitor was transfected to MDA-MB-468 and BT459 cells (Fig. 4c). Next, we co-transfected miR-125a-5p mimic and circ_0001667 3′UTR into breast cancer cells and the dual luciferase system was used to exam the luciferase activity. The data indicated that miR-125a-5p mimic reduced the luciferase activity of circ_0001667 3′UTR WT rather than circ_0001667 3′UTR MUT (Fig. 4d). Remarkably, the luciferase activity was increased when miR-125a-5p inhibitor and circ_0001667 3′UTR was co-transfected to breast cancer cells (Fig. 4e). To confirm the relationship of circ_0001667 and miR-125a-5p, MDA-MB-468 and BT459 cells were treated with circ_0001667 shRNA and the result from qRT-PCR showed that the level of miR-125a-5p was over-expressed when circ_0001667 was silenced (Fig. 4f). To test whether miR-125a-5p affects circ_0001667 expression, miR-125a-5p was introduced into MDA-MB-468 and BT459 cells and the expression of circ_0001667 was not affected (Fig. 4g). Our data confirmed that circ_0001667 functionally interacted with miR-125a-5p and served as a sponge for miR-125a-5p.

### Circ_0001667/miR-125a-5p/TAZ activated Hippo pathway in breast cancer cells

**Fig. 5:**
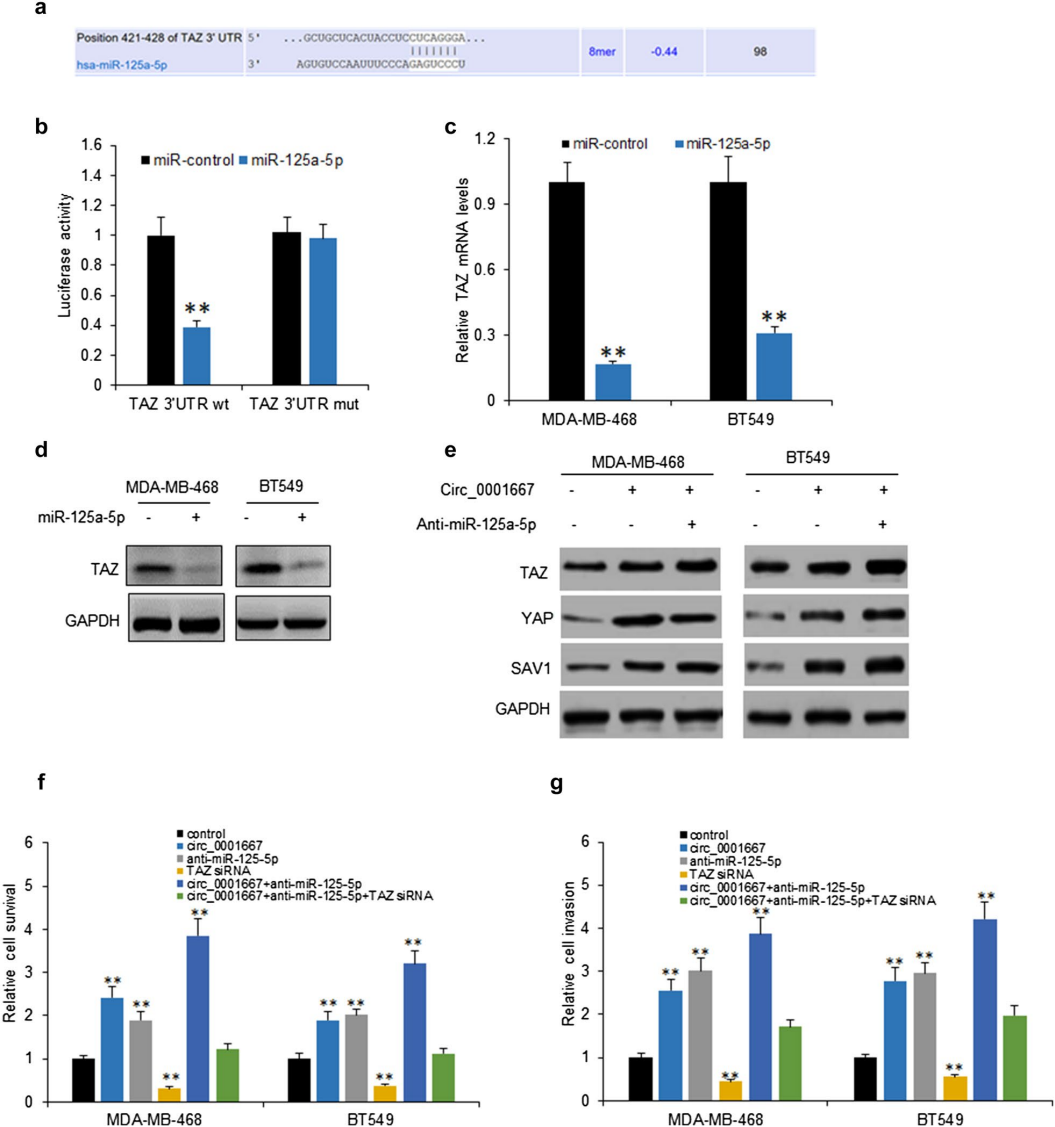
Circ_0001667/miR-125a-5p/TAZ activated Hippo pathway in breast cancer cells. **a** TAZ was a predicted target gene of miR-125a-5p by Targetscan. **b** TAZ 3′UTR activity in MDA-MB-468 cells with miR-125a-5p transfection. **c** TAZ mRNA in MDA-MB-468 and BT549 cells. Cells were transfected with miR-125a-5p TAZ mRNA was analyzed by qRT-PCR. **d** TAZ protein levels in MDA-MB-468 and BT549 cells. Cells were transfected with miR-125a-5p and the expression of TAZ was determined by western blotting. **e** Circ_0001667 promoted YAP, SAV1, TAZ expression. MDA-MB-468 and BT549 cells were transfected with Circ_0001667 shRNA or miR-125a-5p and the expression of YAP, SAV1, TAZ was determined by western blotting. **f** Cell proliferation in MDA-MB-468 and BT549 cells. Cells were transfected with circ_0001667 shRNA, miR-125a-5p or TAZ and cell survival ability was assayed by CCK8. **g** Cell invasion in MDA-MB-468 and BT549 cells. Cells were transfected with circ_0001667 shRNA, miR-125a-5p or TAZ and cell invasion ability was assayed by transwell system. **p < 0.01

To assess the downstream target gene of miR-125a-5p, bioinformatic analysis was used to predict via Targetscan which showed that TAZ was the target gene of miR-125a-5p (Fig. 5a). To further validate the interactions of miR-125a-5p and TAZ, MDA-MB-468 cells were co-transfected with miR-125a-5p and TAZ 3′UTR. The result from dual-luciferase assay showed that miR-125a-5p decreased the luciferase activity of TAZ 3′UTR WT instead of TAZ 3′UTR MUT (Fig. 5b). MiR-125a-5p over-expression in MDA-MB-468 and BT-549 cells dramatically reduced TAZ expression levels by qRT-PCR (Fig. 5c), as well as protein levels (Fig. 5d). In addition, to find the relationship of miR125a-5p and Hippo pathway, MDA-MB-468 and BT-549 cells were transfected with circ_0001667 or miR-125a-5p inhibitors and the western blot result indicated that circ_0001667 promoted the protein expression of TAZ, YAP and SAV1 in the cells with or without miR-125a-5p down-regulation (Fig. 5e). Further cellular functions indicated that circ_0001667 promoted the cell survival and invasion in MDA-MB-468 and BT-549 cells with or without miR-125a-5p down-regulation, and also revised the cellular proliferation or invasion in the cells with TAZ down-regulation (Fig. 5f, g). These findings provided further evidence that circ_0001667/miR-125a-5p/TAZ activated Hippo pathway in breast cancer cells.

### The relationship between circ_0001667, miR-125a-5p and TAZ expression in breast cancer tissues

**Fig. 6:**
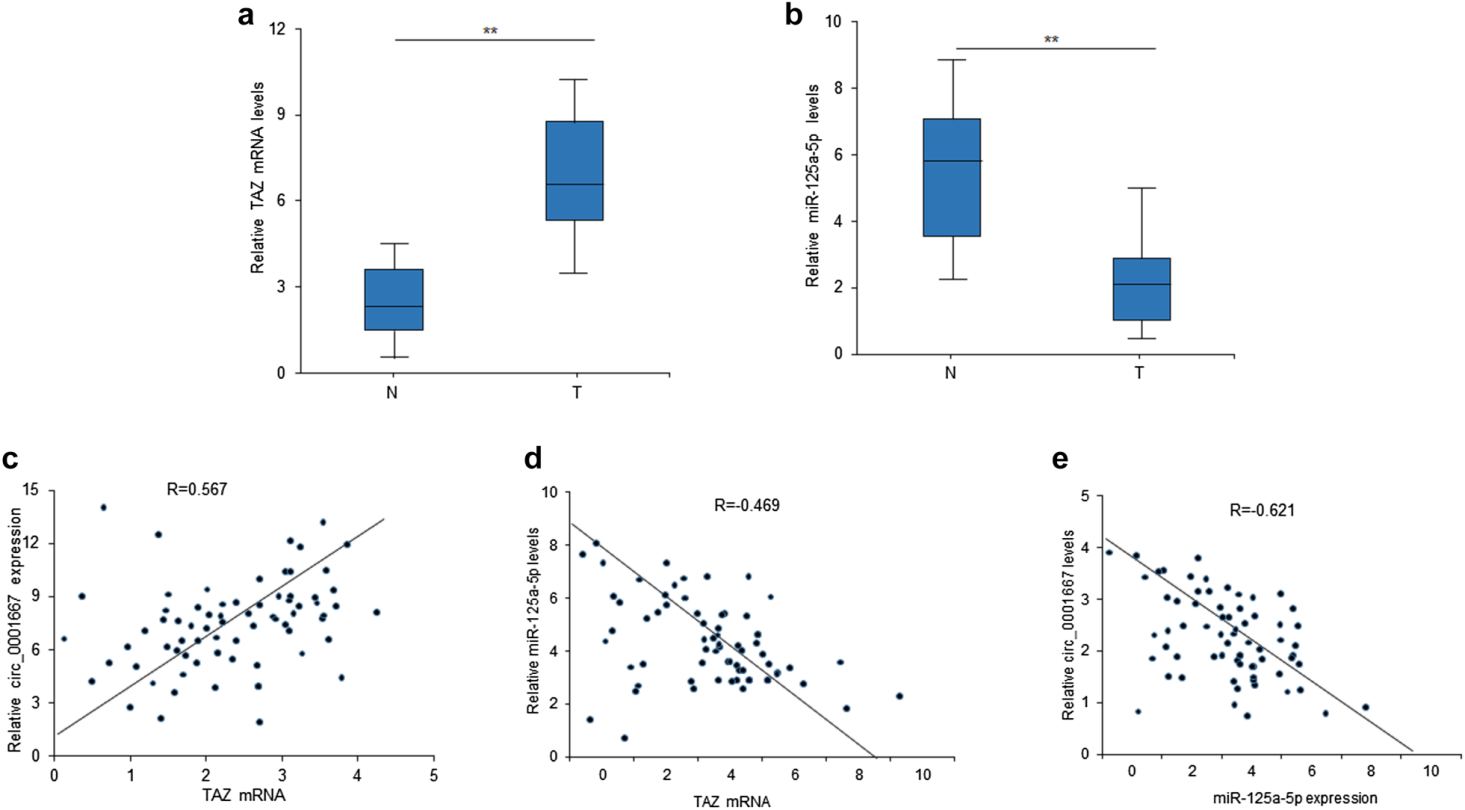
The relationship between circ_0001667, miR-125a-5p and TAZ expression breast cancer tissues. **a** The total RNA was extracted from breast cancer tissues and TAZ mRNA expression was examined by real time RT-PCR. **b** miR-125a-5p expression in breast cancer tissues was examined by real time RT-PCR. **c** Relationship between TAZ mRNA and circ_0001667 expression in breast cancer tissues. **d** Relationship between TAZ mRNA and miR-125a-5p expression in breast cancer tissues. **e** Relationship between miR-125a-5p and circ_0001667 expression in breast cancer tissues. **p < 0.01

To further learn the relationship between circ_0001667, miR-125a-5p and TAZ, we detected the TAZ and miR-125a-5p mRNA levels from breast cancer tissues by qRT-PCR. The result showed that TAZ mRNA was higher in tumor tissues than normal tissues (Fig. 6a). Conversely, the expression of miR-125a-5p in tumor tissues was lower than normal tissues (Fig. 6b). According to the qRT-PCR result, it was demonstrated that TAZ had a positive correlation with circ_0001667 (Fig. 6c) and a negative relationship with miR-125a-5p (Fig. 6d). Moreover, it was also revealed a negative correlation of miR-125a-5p and circ_0001667 (Fig. 6e). Thus, we confirmed the relationship between circ_0001667, miR-125a-5p and TAZ in breast cancer.

## Discussion

Currently, with the intensive study of non-coding RNA, researchers have paid enough attention to circRNAs. CircRNAs exist widely in various organisms and cells [20]. Given their vital role as regulators, circRNAs can be a potential biomarker for disease diagnosis because of its organization and disease-specificity [21]. Multidisciplinary research suggests that the circRNAs play important roles in cancer [22, 23], but not much to breast cancer. So, we hypothesized that circRNAs take participate in the progress and development of breast cancer. In the present study, we found circ_0001667 was up-regulated in breast cancer tissue using circRNA microarray. Further analyses were conducted to study the preliminarily biological function of circ_0001667. Down-regulation of circ_0001667 suppressed cell proliferation and metastasis in MDA-MB-468 and BT549 cells, suggesting that circ_0001667 inhibition may be a promising broad-based strategy for impeding cells proliferation and metastasis in breast cancer.

Bioinformatics analysis, an emerging discipline, was useful for the biologist to explore the underlying mechanism and interrelation of molecules. CircRNA can sponge miRNAs to regulate target genes. We predicted miRNA sponged by circ_0001667 using bioinformatics analysis and the result demonstrated that miR-125a-5p was sponged with circ_0001667. Dual luciferase reporter assay and real-time fluorescence quantitative PCR experiment also confirmed the relation of circ_0001667 and miR-125a-5p. Further bioinformatics analysis revealed the mapping of pathways in cancer mediated by miR-125a-5p and the Hippo pathway had a relationship with circ_0001667/miR-125a-5p.

Hippo pathway takes charge in organ size and tumorigenesis in various animal models [24–31]. TAZ and YAP are critical oncogenes in Hippo pathway [13]. Remarkably, TAZ abundance is increased in invasive breast cancer cell lines and in 20% of breast cancer tissues [32]. Many reports also have demonstrated the function of TAZ in breast cancer. For instance, TAZ was regulated by Ski to suppress breast cancer progression [33]. Synaptopodin-2 (SYNPO2) inhibited YAP/TAZ to suppress triple-negative breast cancer (TNBC) metastasis [34]. Our result also confirmed that TAZ has a critical role in breast cancer. TAZ was predicted and verified as a target gene of miR-125a-5p by Targetscan and dual luciferase assay respectively. The result from western blotting showed that the expression of TAZ was increased under over-expression of circ_0001667 and inhibited on the over-expression of miR-125a-5p. To further investigate the relationship of circ_0001667/miR-125a-5p and TAZ in breast cancer, qRT-PCR was implemented in breast cancer tissues. The experimental data clarified that circ_0001667 promoted TAZ expression and reversely miR-125a-5p suppressed the expression level of TAZ. As expected, we also found that there was a negative correlation between miR-125a-5p and circ_0001667. These findings suggested the roles for both circ-0001667/miR-125a-5p and TAZ in breast cancer.

## Conclusions

In summary, our study showed that circ_0001667 bound miR-125a-5p to regulate TAZ expression and promoted breast cancer cell proliferation and survival via Hippo signal pathway. Taken together, circ_0001667 is a potential breast cancer prognostic marker, as well as a potential therapeutic target to inhibit breast cancer metastasis.

## Methods

### Breast cancer samples

Malignant breast cancer tissues and their adjacent non-malignant tissues were gained from the Affiliated Hospital of traditional Chinese, Medicine Xinjiang Medical University. The informed contents were provided and written for all the patients from the Ethics Boards. The samples from breast cancer patients were collected between 2005 and 2012. The samples were collected and frozen at − 80 °C.

### Cell culture

Cell lines including human breast cancer (MDA-MB-468, MCF-7, T4TD, BT459 and MDA-MB-435) and normal human breast epithelial cells (MCF-10A) were primarily purchased from ATCC. Respectively, MCF-10A was cultured in RPMI1640 growth medium supplemented with 10% fetal bovine serum (Gibco), 50 U/ml of penicillin and 50 μg/ml of streptomycin, EGF and insulin. Other human breast cancer cells were maintained in High-DMEM completed growth medium. All cells were cultured at 37 °C and 5% CO_2_.

### CircRNA, siRNAs and transfection

For lentivirus and plasmid DNA transfection, cells were transfected with specific shRNA duplexes targeting circ_0001667 construct using Lipofectamine 2000 (Invitrogen) according to the manufacturer’s instruction. To confirm the cellular function of miR-125a-5p, cells was transfected with miR-125a-5p mimics or miR-125a-5p inhibitor using Lipofectamine 2000 (Invitrogen). TAZ siRNAs and its control were obtained from RiboBio (Guangzhou, China) and also transfected to cell using Lipofectamine 2000 (Invitrogen).

### Cell proliferation

CCK-8 (Beyotime, Haimen, China) was used to detect the cell proliferation according to the manufacturer’s protocol. About 5 × 10^3^ cells were plated per well in 96-well plates with high DMEM completed medium. The absorbance was measured at 450 nm through microplate reader (BioTek) from 0 to 5 days. All experiments were repeated at least three times independently.

### Cell migration and invasion assay

Transwell plates (8 μm pore size, 6.5 mm diameter; Corning, USA) precoated with Matrigel Basement Membrane Matrix (coating concentration: 1 mg/ml; BD Biosciences, Franklin Lakes, NJ) were used to perform the cell invasion assay (MDA-MB-468 and BT459). 1 × 10^4^/well cells were seeded into the upper chamber of transwell filter on a 24-well plate in which the upper chamber medium contained 1% FBS and 10% FBS medium was added to the lower well of the plate [35]. After 72 h of incubation, the transwell plates were washed and the lower side cells from the upper side of the filters were fixed with methanol. Then, the cells were stained with Giemsa and the cells were counted in five randomly chosen fields per transwell filter under a microscope. Migration assays were performed with the same procedure, except that the transwell chamber inserts were not coated with Matrigel, and medium containing 1% FBS was used for the cell suspensions. Each experiment was repeated three times.

### Dual luciferase reporter assay

For luciferase reporter assay, circ_0001667 Dual-luciferase vectors (Gene Pharma) were used to construct dual luciferase reporter plasmids. Sequences of miR-125a-5p and circ_0001667 were separately cloned into the vectors. MDA-468 cells were co-transfected with wild-type circ_0001667 or mutated type and miR-125a-5p mimics or negative control using Lipofectamine 2000 (Invitrogen). After induction for 48 h, luciferase activity was assessed using the dual-luciferase reporter kit (Promega, Madison, WI, USA). The relative firefly luciferase activity was normalized to Renilla luciferase activity.

### RNA extraction and real-time PCR analysis

Total RNA was extracted from cells and tissues using TRIzol regent (Invitrogen, Carlsbad, CA, USA) according to the manufacturer’s specification. cDNA was reversely transcribed from total RNA by Prime Script TM RT reagent Kit with gDNA Eraser (TaKaRa, Dalian, Liaoning, China). The qRT-PCR was performed in triplicate using the SYBR- Green PCR Master Mix kit (Takara, Japan) on a Step One Plus real time PCR system (Applied Biosystems, Foster City, CA, USA). The analyses were based on the comparative Ct method (2−ΔΔCt) with GAPDH or 18SRNA as the reference genes for microRNA and other genes, respectively. All the primers used in the study were ordered from Sangon (Shanghai, China).

### Western blotting

Cells were lysed in cold RIPA lysis buffer including a protease inhibitor cocktail (Sigma-Aldrich, USA). The protein concentrations were measured by a bicinchoninic acid (BCA) protein assay kit (Pierce Biotechnology, Rockford, IL, USA). Equal protein concentrations were loaded and separated by SDS-PAGE and then electrophoretically transferred to PVDF membranes (Pall, New York, NY, USA). After blocking in 5% skimmed milk powder in Tris-based saline with Tween 20 for 1 h at room temperature, the targeted primary antibodies (cell signaling technology, USA) were incubated in the PVDF membranes at 4 °C overnight. The membranes were further incubated with a secondary antibody for 2 h, after washed in Tris-based saline with Tween20. At last, the membranes were scanned with Odyssey infrared imaging system. All primary antibodies come from cell signaling technology, USA. The secondary antibodies and B-actin antibodies come from Kangcheng Bio-tech, China.

### Statistical analysis

All statistical analysis was performed using the SPSS 15.0 statistical software.

## Data Availability

The datasets used and/or analysed during the current study are available from the corresponding author on reasonable request.
